# Treatment of Fractures of the Lower Jaw

**Published:** 1858-12

**Authors:** W. W. Allport

**Affiliations:** Dentist


### TREATMENT OF FRACTURES OF THE LOWER JAW.

BY W. W. ALLPORT, DENTIST.

Editors of the Chicago Medical Journal.—In the August number of your Journal, Dr. M. O. Heydock reported a case of fracture of the lower jaw, at its neck.

This was a case of peculiar interest, from the fact that it had been under treatment for two weeks, and it was found that no ordinary bandages or appliances would keep it from swaying to one side; of course a deformity would have been the result of such practice. At the expiration of this time, Dr. H. called on me, with the patient, and I constructèd for her two plates, as described in said report, and proved to be just what was required for the successful treatment of the case.

On the 19th of September, Mr. A. G. received a blow upon the right side of the lower jaw, which fractured it through the median line. The case was seen by Professor Freer, who applied the ordinary bandage, used in such cases, but found it would be impossible to secure a satisfactory result in this way, and at once placed the patient in my hands.

When the case came to me, I found the right side of the jaw depressed about eight lines below the left, and the muscles acting with so much force, that it was found difficult to bring the two sides into position, so that the lower teeth would articulate with the upper correctly, and when in position, difficult to keep them so.

With the aid of an assistant, the fracture was adjusted, and an impression taken in wax of the lower teeth, also one impression of the upper teeth, and from these, plates are swaged up, similar to those used in the case of Mrs. H. These being brought into proper position on the teeth, were removed and soldered together. Thin layers of gutta percha were then warmed in hot water, and placed inside of each plate and introduced into the mouth, as warm as the patient could bear;—the jaws were pressed firmly together, until the teeth articulated perfectly, *through the plates*, which had been filed away for the free egress of the ends of the teeth, and were held thus until the gutta percha had been thoroughly cooled by the application of cold water. This done, the patient could not open his mouth, and of course the jaw was held fimly in place, which rendered union perfect, and a natural articulation of the teeth. The plate was removed on the 18th day.

In the case reported by Dr. Heydock, it was supposed that the separation of the upper front teeth, so as to admit of a bar or wedge being passed between them, from the plate on the lower jaw, was of service; but from this case it would seem that the wedge can be dispensed with, for the gutta percha insinuates itself into all separations, and adapts itself to all the irregularities and inequalitiesof the teeth, in such a way, that the patient can not move the lower jaw, and of course it is held firmly wherever it is placed, making this appliance equally serviceable whether the front teeth are separated or not.

The fact that the lower jaw hangs loose, and its muscular attachments draw it in various directions, renders the treatment of the fracture of this one far from being the simplest in surgical practice. In fact, I think that any experienced surgeon will bear witness to the correctness of the statement, that there have been as few cases of perfectly satisfactory results, in the treatment of fracture of the lower jaw, as in almost any other class of broken bones. But if the upper jaw be made the foundation, to which the lower jaw can be immovably attached, by the application of plates constructed as above mentioned, the treatment of these fractures can be made comparatively simple and much more satisfactory.

